# Pharmacotherapeutics Applications and Chemistry of Chalcone Derivatives

**DOI:** 10.3390/molecules27207062

**Published:** 2022-10-19

**Authors:** Jagjit Singh Dhaliwal, Said Moshawih, Khang Wen Goh, Mei Jun Loy, Md. Sanower Hossain, Andi Hermansyah, Vijay Kotra, Nurolaini Kifli, Hui Poh Goh, Sachinjeet Kaur Sodhi Dhaliwal, Hayati Yassin, Long Chiau Ming

**Affiliations:** 1 PAPRSB Institute of Health Sciences, Universiti Brunei Darussalam, Gadong BE1410, Brunei; 2 Faculty of Data Science and Information Technology, INTI International University, Nilai 71800, Malaysia; 3 Faculty of Engineering, Universiti Teknologi Malaysia, Skudai 81300, Malaysia; 4 Centre for Sustainability of Ecosystem and Earth Resources (Pusat ALAM), Universiti Malaysia Pahang, Kuantan 26300, Malaysia; 5 Department of Pharmacy Practice, Faculty of Pharmacy, Universitas Airlangga, Surabaya 60115, Indonesia; 6 Faculty of Pharmacy, Quest International University, Ipoh 30250, Malaysia; 7 Faculty of Integrated Technologies, Universiti Brunei Darussalam, Gadong BE1410, Brunei

**Keywords:** antimicrobial agent, infectious disease, cancer, cardiovascular disease, health benefits, pharmacology

## Abstract

Chalcones have been well examined in the extant literature and demonstrated antibacterial, antifungal, anti-inflammatory, and anticancer properties. A detailed evaluation of the purported health benefits of chalcone and its derivatives, including molecular mechanisms of pharmacological activities, can be further explored. Therefore, this review aimed to describe the main characteristics of chalcone and its derivatives, including their method synthesis and pharmacotherapeutics applications with molecular mechanisms. The presence of the reactive α,β-unsaturated system in the chalcone’s rings showed different potential pharmacological properties, including inhibitory activity on enzymes, anticancer, anti-inflammatory, antibacterial, antifungal, antimalarial, antiprotozoal, and anti-filarial activity. Changing the structure by adding substituent groups to the aromatic ring can increase potency, reduce toxicity, and broaden pharmacological action. This report also summarized the potential health benefits of chalcone derivatives, particularly antimicrobial activity. We found that several chalcone compounds can inhibit diverse targets of antibiotic-resistance development pathways; therefore, they overcome resistance, and bacteria become susceptible to antibacterial compounds. A few chalcone compounds were more active than conventional antibiotics, like vancomycin and tetracycline. On another note, a series of pyran-fused chalcones and trichalcones can block the NF-B signaling complement system implicated in inflammation, and several compounds demonstrated more potent lipoxygenase inhibition than NSAIDs, such as indomethacin. This report integrated discussion from the domains of medicinal chemistry, organic synthesis, and diverse pharmacological applications, particularly for the development of new anti-infective agents that could be a useful reference for pharmaceutical scientists.

## 1. Introduction

**Figure 1:**
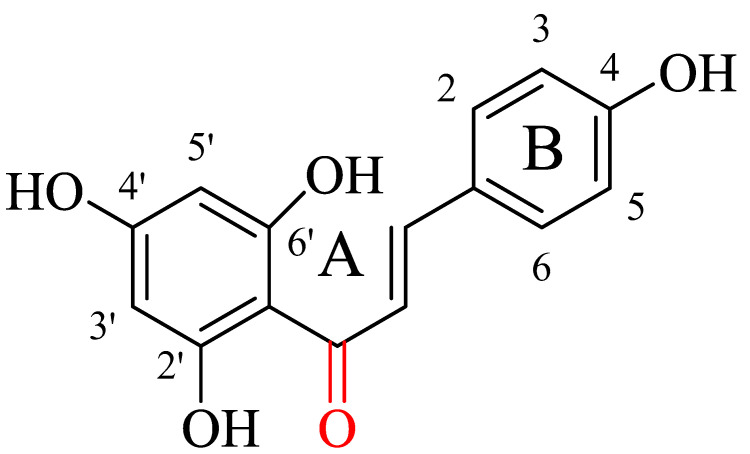
Core structure of chalcone molecule.

Chalcone is a collective group of ketones (flavonoids) that has a three-carbon α,β-unsaturated carbonyl group attached to two aromatic rings, namely rings A and B (Figure 1). The numbering system of chalcone shown in Figure 1 is followed throughout this article. Other chemical names of chalcone include benzyl acetophenone or benzylideneacetophenone. They are produced by certain plant species such as *Angelica*, *Glycyrrhiza*, *Humulus*, and *Scutellaria* as precursors to the biosynthesis of flavonoids and isoflavonoids and intermediates to the synthesis of heterocyclic compounds with biologically interesting properties such as pyrazolines, isoxazoles, cyanopyridines and pyrimidines.

Chalcone is part of plants’ most prominent class of secondary metabolites. This is used in plant defense mechanisms to combat reactive oxygen species for the plant to survive and prevent molecular damage as well as damage caused by microorganisms, insects, and animals [1]. Chalcone can also be chemically synthesized in the laboratory using the Claisen Schmidt or aldol condensation reaction [2]. Chalcone has been reported to exert multiple beneficial properties, such as anti-inflammatory, antibacterial, antifungal, antidiabetic properties, and anticancer activities. It also improves vision, memory, joint and muscle discomfort, liver and kidney function, sleep, prevents cancer, strengthens the immune system, and beautifies skin and hair. [3,4,5].

Chalcone and its derivatives have shown an inhibitory effect against methicillin-resistant *Staphylococcus aureus* (MRSA) [6] due to the presence of −OH groups in the B ring and the lipophilicity of the A ring (Figure 1) [7,8]. A combination of Chalcones and antibiotics (i.e., oxacillin) has also shown a synergistic effect in treating MRSA infections [9,10,11,12].

Additionally, some chalcone derivatives exhibited antifungal activities, particularly against *Microsporum gypseum* [13,14,15,16]. They inhibit the β (1, 3)-glucan and chitin synthases responsible for the formation and normal functioning of the fungal cell wall [17,18]. Some chalcone compounds have shown superior antifungal effects compared to ketoconazole, a broad-spectrum oral antifungal agent [13]. Chalcone compounds are also effective inhibitors of inflammatory enzymes, such as cyclooxygenase (COX), lipooxygenase (LOX), interleukins (IL), and prostaglandins (PGs) [19]. The most researched novel series of chalcones revealed their potent inhibitory effects on nitric oxide (NO) formation and the release of glucuronidase and lysozyme, which are responsible for inflammatory responses [20]. Heterocyclic rings and methoxy substitutions on the attached rings of chalcones contribute to the anticancer properties of such chalcone compounds [21,22,23]. The investigated chalcone compounds: isoliquiritigenin, flavokawain, and xanthohumol, showed cytotoxic and apoptosis induction that promotes antitumor activities [23]. However, the mechanisms of the compound are unclear, causing uncertainty about chalcones’ astounding abilities. It is a compound that has attracted the attention of many scientists due to its plethora of therapeutic efficacy.

Numerous studies have been conducted to explore the pharmacological properties of chalcone and its derivatives in recent years. Even though there are a few reviews available, such as antiviral [24,25], preclinical studies [4], synthesis chalcone [26], and antidiabetic [27], comprehensive reviews focusing on the overall purported health benefits, including molecular mechanisms of pharmacological actions of chalcone and its derivatives evaluating the recent progress of therapeutical applications is insufficient. Therefore, herein, we reviewed the research findings to discuss the synthesis of chalcone and its derivatives and assess their pharmacotherapeutic efficacy, focusing on the recent experimental studies. This report integrated discussion from the domains of medicinal chemistry, organic synthesis, and diverse pharmacological applications, particularly for the development of new anti-infective agents that could be a useful reference for pharmaceutical scientists.

## 2. Chemistry of Chalcone and Its Derivatives

Chalcone and its derivatives have been long used in various traditional medicine systems, including homeopathic and Chinese medicine. They are traditionally prepared by the reaction of benzaldehydes and active methylene ketones under homogeneous conditions using the Claisen-Schmidt condensation and a more recent invention known as the aldol condensation [28]. However, recent discoveries of methods for producing chalcones provide different advantages depending on the type of catalyst, solvent, base, and reaction conditions [29].

### 2.1. Claisen Schmidt Condensation

**Figure 2:**
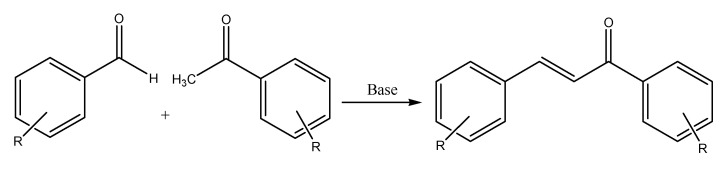
Claisen-Schmidt condensation reaction.

The Claisen Schmidt condensation reaction involves an aldehyde with the carbonyl group without hydrogen atoms in the α-position, and a ketone, using a heterogeneous acid catalyst to produce the desired α,β-unsaturated ketone. This is one of the methods of synthesizing chalcone in the laboratory (Figure 2) due to the equimolar quantities of acetophenone and benzaldehyde. Claisen Schmidt condensation uses an aqueous-alcoholic alkali with a (concentration of 10 to 60%) to catalyze the reaction between acetophenone and benzaldehyde by dehydration [30]. The reaction can take place either for 12–15 h at a temperature of 50 °C or for one week at room temperature (20–25 °C).

The heterogeneous acid catalyst is useful in producing chalcones because of the increased purity of end-products, decreased amounts of undesired products, reduced reaction time, and cost-effective procedure. Ionic liquids (ILs) are prepared using this condensation reaction but use a multi-sulfonic acid group ion liquid as the catalyst. ILs have gained interest due to their high catalytic activity, small catalyst usage, easy filtration, recyclable catalyst, and constant catalytic activity (Figure 2). However, the product yield will be decreased due to the reaction conditions, which promotes the Cannizzaro reaction. This redox reaction produces primary alcohol and carboxylic acid from two aldehyde molecules.

### 2.2. Aldol Condensation

**Figure 3:**
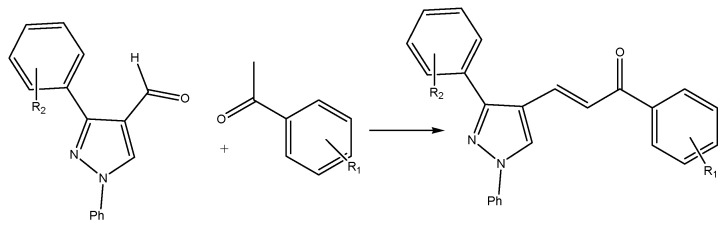
Heterocyclic ring-containing chalcone using a phase transfer catalyst.

Aldol condensation is another synthetic method commonly used after the Claisen-Schmidt condensation. The aldol condensation reaction (Figure 3), or the solid-state reaction, replaces aldehydes with benzylidene-diacetate and uses heat (200–350 °C) and a base such as potassium hydroxide as a catalyst for the reaction between the two compounds. It uses calcium, barium or strontium hydroxides or carbonates as catalysts in a liquid mixture containing water with a low boiling point that can perform distillation at a constant temperature. Other synthetic reactions can increase the reaction rate using microwave radiation without solvents; it also provides fluorescence emission profiles that can be used as biological markers.

Reacting a ketone with an aldehyde (with the carbonyl group-containing no hydrogen atoms in the α position with an acidic heterogeneous catalyst-activated carbon) will reduce the reaction time, cost, and impurities in the final products. Another technique involves the phase transfer method for synthesizing heterocyclic ring-containing chalcones, which incorporates a third ring into the chalcone’s skeleton chain. The starting constituents used are 1-phenyl-3-aryl-4-formyl pyrazole and acetophenone, with the catalyst being tetrabutylammonium bromide in the presence of an inorganic alkaline solution; the reaction is performed under microwave radiation (Figure 3).

### 2.3. Synthesisand Chemistry

Another method uses plants such as *Sedum jinianum*, *S. plumbizincicola*, *S. alfredi* and *Potentilla griffithii* with a high concentration of metals such as zinc, copper, cadmium, and magnesium, and at least one of the metal elements is used as a catalyst. They are first heated and treated with acid, then filtered and purified to be attached to a carrier, becoming a metal catalyst in synthesizing chalcones. Although this method promotes less pollution, it is unconventional to extract metal from these plants to be used as mere catalysts.

A different method uses a fluorine-containing biphasic catalyst as a result of reacting 4-dimethylamino pyridine with fluorinated alkyl iodide to react benzaldehyde with acetophenone at 50 to 100 °C for 1 to 3 h. The reacted compounds were cooled, filtered, distilled, and recrystallized with more than 99% purity. This invention requires an easy processing method, nature-friendly, low synthesis cost, and ease of re-obtaining the fluoric catalyst.

There are methods to synthesize hydroxyflavones that use soluble resin, specifically polyethylene glycol (PEG) that reacted with benzyloxy-2-hydroxy-acetophenone to be used in a reaction with benzaldehyde with a base as a catalyst. The advantages of this method are high accessibility to the reactant compounds and high purity percentage.

Another popular method is the one-pot synthesis. In this process, a direct reaction in one step is utilized to prepare inorganic components, while the organic component operates as a surface capping material or template. This method shortens the time to separate and purify the products and increases the product yield. Another technique of one-pot synthesis is slowly adding primary alcohol with chromium oxide (CrO_3_) to produce furochalcones, or the addition of cheap catalysts, which are copper salt, 2,2′-bipyridine, and 2,2,6,6-Tetramethylpiperidinyloxy (TEMPO) kept at a temperature of −10 to 100 °C for 10 to 96 h to produce a milder reaction.

A different synthesis method to produce the α,β-unsaturated carbonyl system that does not require condensation reaction offers more direct response with fewer vigor conditions, a cheaper cost of reactants, a more straightforward operating system, and a higher yield of products. The raw materials are the halogenated aromatic hydrocarbons with a ketone in the carbon skeleton and aromatic alkynes, using an alkali, a phosphine ligand, and palladium as catalysts, at a temperature of 60 to 150 °C [31,32].

### 2.4. Approach to Design of Chalcone Derivatives from the Natural Sources

Different approaches have been used to design chalcone derivatives, as outlined below:

#### 2.4.1. Isoliquiritigenin

**Figure 4:**
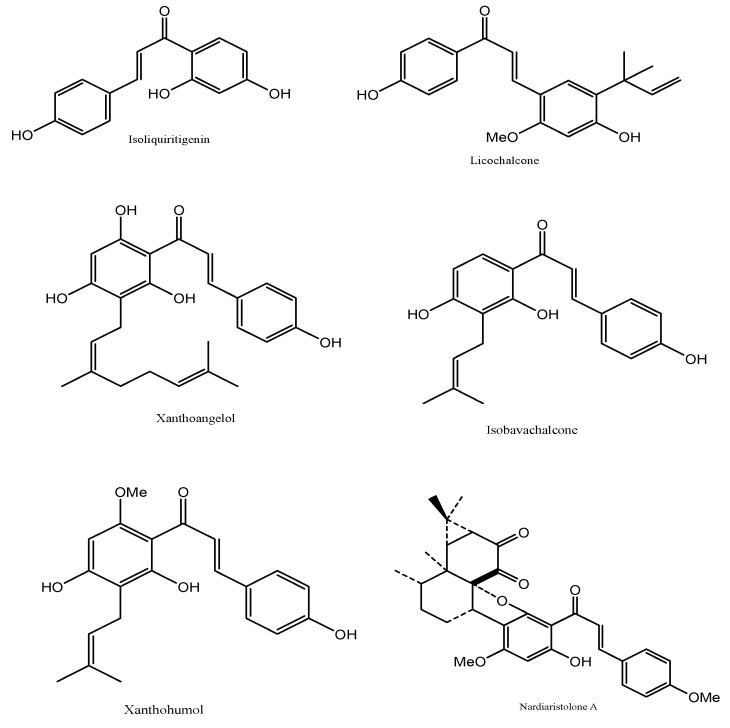
The derivatives of Chalcones from natural sources.

It can be extracted from the plant *Radix Glycyrrhizae*. Isoliquiritigenin is used in cosmetics due to its beneficial effects on the skin, including treatment of skin conditions such as acne, eczema, and irritation, as well as desirable effects, such as skin whitening, anti-aging, and eye drop preparations. There have been claims that isoliquiritigenin can activate the GABA_A_ receptor, bind to the γ-subunit and act as a positive allosteric modulator that elicits similar effects as a benzodiazepine. It also prevents and treats cardio-cerebrovascular diseases [33] (Figure 4).

#### 2.4.2. Licochalcone A

It is present in high concentrations in the plant *Glycyrrhiza inflate* [34]. Licochalcone A and isoliquiritigenin compounds are used in the cosmetic industry; they are both used in acne treatment and skin whitening. Licochalcone A is also used to prepare skin toner and hair cosmetics. The compound in essential oils produces bath salts to clean pores in the skin, control sebum production and retain skin moisture. Licochalcone A is also used for the treatment, improvement, and prevention of adenosine 5′ monophosphate-activated protein kinase (AMPK)-related diseases, an enzyme involved in metabolism, specifically for lipids [34]. Influenza virus infection-related diseases were treated for other conditions in which licochalcone A was used [35,36,37] (Figure 4).

#### 2.4.3. Xanthoangelol

It is a major component of the plant *Angelica keiskei*. Xanthoangelol is an isoprenyl-chalcone compound with antioxidant properties that are used to prevent diseases involving lipid metabolism or inflammation [38] (Figure 4).

#### 2.4.4. Isobavachalcone

It can be isolated from *Psoralea corylifolia* or *Piper longum* fruits. It belongs to the same chalcone family as xanthoangelol but has different properties [39]. It inhibits melanin formation causing skin whitening [40]. Isobavachalcone is also used to reduce nerve inflammation and inhibit cholesterol absorption [41]. Other uses of isobavachalcone prevent and control diseases [42] (Figure 4).

#### 2.4.5. Xanthohumol

It is present in the *Humulus lupulus* and belongs to the prenylated chalcone family. Its antioxidant property is a ‘broad spectrum’ cancer chemo-preventive agent. Along with hydroxytyrosol, it is used as a nasal spray for viral infection, allergic reactions, or vasomotor rhinitis of the nasal mucosa [43]. Xanthohumol has demonstrated the inhibitory activity of the enzyme α-glucosidase, an enzyme responsible for carbohydrate metabolism. Therefore, it is used in metabolic diseases such as diabetes and other diseases such as AIDS, osteoporosis, and malignant tumors [44,45] (Figure 4).

#### 2.4.6. Nardoaristolone A

It can be extracted from *Nardostachys chinensis* and classified as terpenoid chalcones [46]. Like any other chalcones, it has been used to treat different skin cancers. It has systemic effects such as increasing the red blood cell count and aids in small bowel movements. Nardoaristolone A has been used in medications for tuberculosis and endometrium cancers [1,45] (Figure 4).

### 2.5. Role of Chalcone Moiety in Synthesis of Derivatives

The chalcone moiety can be used to produce other chalcone derivatives such as cyanopyridines, pyrazolines, isoxazoles, and pyrimidines with different heterocyclic ring systems. These derivatives containing hydroxyl, ether, acid, or amino groups have diverse functionality and can be used to produce more complex chalcones. Aminochalcones, a chalcone moiety, can produce benzothiazole chalcones and other derivatives through alkylation, hydrolysis, and esterification or amide formation. Chalcone derivatives can be produced through the Phase Transfer Catalysis through alkylation, using oxygen or sulfur.

Chalcone derivatives can also be produced using the α,β-unsaturated system by substitution of the functional group at the two positions, and dihydrochalcones can be produced by reducing the double bond in the saturated system; these derivatives will be used in the synthesis of pyrazoles and flavonoids as well as other heterocyclic compounds.

A chalcone derivative which is 3-phenyl-1-(4-methyl) phenyl-2-bromo-propylene-1-one, can be produced by reacting ρ-toualdehyde with acetophenone using an inorganic base as a catalyst in an aldol condensation reaction, and the products react with a halogen in an addition reaction followed by an elimination reaction with an inorganic base. Another method also used a heterogeneous catalyst (hexagonal boron nitride h-BN) that is hydrogenated and has a Frustrated Lewis Pair (FLP)-type electronic structure that produces 100% yield.

**Figure 5:**
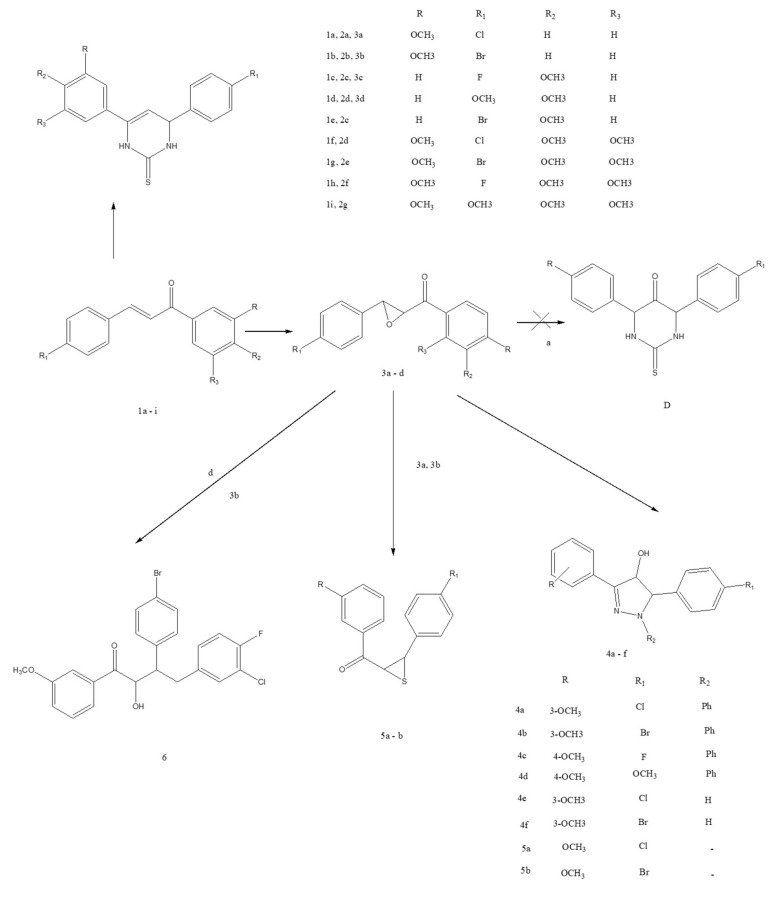
Chemical reactions from Chalcones derivatives.

Another method to obtain dihydrochalcones, specifically phloretin, used bacterial or plant chalcone isomerases and enolate reductase as a catalyst. An electrolytic hydrogenation method using hydrogen, which becomes active by electrolysis of water, is needed, followed by an addition reaction with a ketone without a catalyst to obtain neohesperidin dihydrochalcones. Dihydrochalcones and quinazolinyl derivatives can be produced by substituting carboxylic or nitro groups at the β carbon of the carbon skeleton of chalcones, respectively [47] (Figure 5).

#### 2.5.1. Semi Synthetic Derivatives of Chalcones

**Figure 6:**
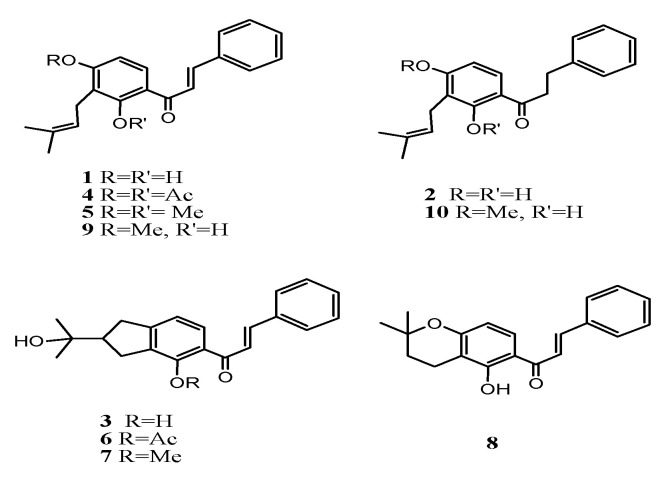
Semisynthetic derivatives of chalcones.

Chalcone isocordoin and its semisynthetic derivatives were tested for Anti-inflammatory and anti-hypersensitive effects in mice (Figure 6).

#### 2.5.2. Characterization of Chalcones

The structure of the synthesized chalcones can be characterized by IR, NMR and mass spectroscopy.

#### 2.5.3. UV Spectrum

The UV spectrum of chalcones consists of two essential absorption band: band I and relatively a minor band, band II. In chalcones, band I usually appears in 340–390 nm, although a minor inflection or peak often occurs at 300–320 nm. Band II appears in 220–270 nm.

#### 2.5.4. IR Spectrum

In the IR spectra of chalcones asymmetric and symmetric stretching vibrations of the aromatic C–H bonds are seen at 3120–3080 cm^−1^ and 3060–3040 cm^−1^ ranges with two low intensity bands. C–H stretching band of the =C–H group is observed at 3030–3010 cm^−1^. The bands at 1610–1570 cm^−1^ are assigned to the vibrations of the aromatic ring. The inplane deformation of the =C–H bond is appeared as broad weak band at 1460–1430 cm^−1^. The carbonyl stretching vibrations for the enones (=C–C=O) can be found between 1650 and 1685 cm^−1^.

#### 2.5.5. NMR Spectrum

The _1_H-NMR spectrum of double bonds of chalcones were seen at 5.4 and 6.1 ppm. The aromatic regions were observed at 6.9–8.1 ppm.

In _13_C-NMR spectrum of chalcones, the carbonyl carbon usually appears between δ 186.6 and 196.8. The α- and β- carbon atoms with respect to the carbonyl group give characteristic signals between δ 116.1–128.1 and δ 136.9–145.4 respectively.

#### 2.5.6. Mass Spectrum

Basic fragmentation pathways of chalcones are obtained by loss of the phenyl group from the A or B ring, and loss of CO.

## 3. Basic Fragmentation Pathways of Chalcones Are Obtained by Loss of the Phenyl Group from the A or B Ring, and Loss of CO. Pharmacotherapeutic Activities

**Figure 7:**
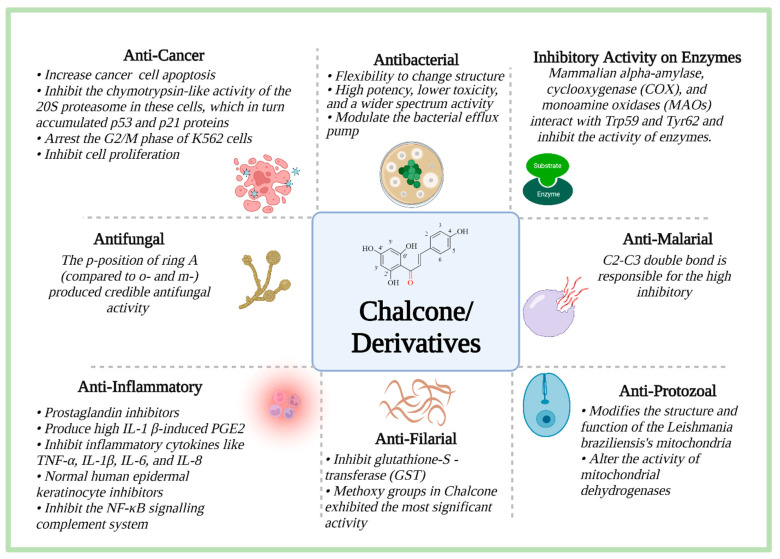
Pharmacological properties of chalcone and its derivatives (Information was sourced from references [19,48,49,50,51,52,53].

Chalcone and its derivatives have shown diverse pharmacological activities. A summary of different pharmacological properties with their salient mechanisms of action is shown in Figure 7. The pharmacophore responsible for various activities changes depending on the activity. The details of these functions have been discussed comprehensively in the following sections.

### 3.1. Anti-Bacterial Agent

Chalcone is an antibacterial agent with moderate to high activity due to the presence of the reactive αβ-unsaturated system. Their flexibility to change their structure by incorporating different types of substituent groups into the aromatic ring can potentially achieve a higher potency, lower toxicity, and a wider spectrum of antibacterial activity [54].

Many chalcone derivatives showed potential antibacterial activities against different pathogenic strains, including antibiotic-resistant bacteria. A summary list is shown in Table 1. Several chalcones were significantly potential than the standard antibiotics (Table 1). For example, compounds **1**–**4**, **6**–**8**, **11**–**13**, **15**–**19**, **21**–**29** (Table 1) have shown strong antibacterial activity against Gram-positive bacteria *Staphylococcus aureus* and *Enterococcus faecalis*, and against Gram-negative bacteria *Escherichia coli* and *Salmonella enterica* [55]. Gram-positive bacteria were more susceptible to cationic molecules than Gram-negative bacteria. For example, compounds **1**–**4** showed the highest activity (MIC ranged 1–2 µg/mL) against Gram-positive bacteria, whereas MIC ranged 2–8 1–2 µg/mL for Gram-negative bacteria. The hydrophobicity of the alkyl chain was responsible for varying strength of antibacterial potentiality. Compounds **7** with medium hydrophobicity exhibited the highest activity against the tested bacteria; however, compounds **6**–**10** with different alkyl chain lengths showed different antibacterial sensitivity. The results reported in Table 1 demonstrated that increasing alkyl chain length (i.e., compounds **8**–**10**) causes decreasing antibacterial activity. Since the long hydrophobic chain has an aggregation tendency, this might cause this decreasing antibacterial activity [56]. Another study reported that 31 compounds, among them, compounds **47**, **50**, and **51** (Table 1), were more active against the tested bacteria, *B. cereus*, *E. coli*, *P. aeruginosa*, and *S. aureus* [57]. Another study tested both sulfones and bisulfones chalcones (**11** compounds) for their antibacterial activity against Gram-positive strains *B. subtilis* and *S. aureus* and Gram-negative strains *P. aeruginosa* and *S. typhimurium* [58]. In this study, in comparison to standard antibiotics Ampicillin and Kanamycin, compound **61** was slightly better against *B. subtilis* and compounds **65**, **66**, and **67** were significantly potential against *S. typhimurium*. In another study using monomeric chalcone compounds, there was higher antibacterial activity in Gram-positive bacteria than the Gram-negative bacteria [59]. A screening method of chalcone derivatives against *S. aureus*, *B. subtilis*, *E. coli*, and *P. aeruginosa* showed increased lipophilic area, and the smaller molecular size of chalcones increased their antimicrobial activity [60].

Chu et al. [55] reported the antibacterial activity of several cationic Chalcones against antibiotic resistance strains, including nine clinical isolates of MRSA, 12 clinical isolated of KPC-2-producing *Klebsiella* pneumoniae (KPC), and 12 clinical isolated of New Delhi metallo-β-lactamase 1 (NDM-1)-producing *Enterobacteriaceae*. A few selected molecules (compounds **1**–**4**, **7**, **12**, **16**–**19**, **26**, and **28**) were tested against MRSA and found MIC values of 0.25–32 μg/mL (Table 1). Among these, compound **7** showed the lowest MIC 0.25 μg/mL, and exhibited the highest activity against clinical isolates 6 of MRSA. The MICs of the other compounds were primarily 0.5–8 μg/mL [55].

For KPC, compounds **17**, **26**, and **28** exhibited the best antibacterial activity (MIC 2 μg/mL), which was equivalent to vancomycin. However, compound **3** showed average activity MIC ranging from 4–8 μg/mL. The MIC for NDM ranged mainly from 1–16 μg/mL; however, among them, compound **28** exhibited the highest activity (MIC: 2–8 μg/mL) [55]. Overall, compound **28** was highly effective against KPC and NDM, whereas compound **7** was for MRSA. Furthermore, the antibacterial activity of the tested molecules against NDM was better than the KPC.

### 3.2. Antibacterial Mechanisms

**Figure 8:**
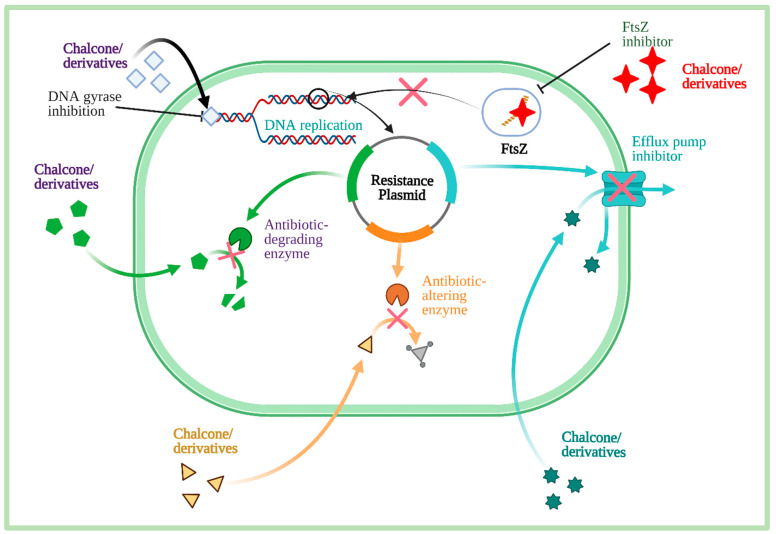
Antibacterial mechanisms of chalcone and its derivatives. Antibiotic resistance bacteria inhibit the function of antibiotic or antimicrobial agents by activating the efflux pump, modifying the active site of the enzyme, and degrading the enzyme. However, chalcone and its derivatives inhibit or block the functions of resistance plasmids through different mechanisms. Additionally, they inhibit DNA gyrase, DNA replication, and FtsZ. Therefore, bacteria cannot cell divide, and ultimately the bacteria die (Information was sourced from reference [61].

Chalcone and its derivatives have the potential to target diverse receptors. This diverse inhibition process includes (i) efflux pump inhibitor (EPI), (ii) type II fatty acid biosynthetic pathway (FAS-II), (iii) DNA replication, (iv) filamentous temperature-sensitive mutant Z (FtsZ), (v) virulence factor, and (vi) protein tyrosine phosphatases (PTPs). A schematic representation of the antibacterial mechanisms of chalcone and its derivatives is shown in Figure 8. The details mechanisms can be found elsewhere (see review [5]).

Chalcones were the potential to exhibit cytoplasmic membrane depolarization and inner membrane permeabilization. Compounds **1**–**4**, **7**, **11**, **12**, **16**–**19** were used to evaluate the ability of the cytoplasmic membrane depolarization and the inner membrane permeabilization against *S. aureus* and *E. coli* to understand their mode of action [55]. The highest cytoplasmic membrane depolarization activity of *S. aureus* was exhibited by compounds **3**, **7**, and **18**, whereas compounds **11**, **1**, and **7** showed for *E. coli*. Both *S. aureus* and *E. coli* cytoplasmic membrane depolarization was at least level by the other compounds.

The ability of bacterial efflux pumps to rapidly export antibacterial drugs makes them sustainable against drugs. Even though the origin of antibiotic resistance is numerous and complex, efflux pumps are one of the critical class of resistance determinants [62,63,64,65,66,67,68,69]. About 117 Chalcone molecule was tested against the NorA efflux pump of *S. aureus* and found at least 20 effective compounds that were able to inhibit this efflux pump. However, five compounds were active, and among them, two compounds, 4-phenoxy-4′-dimethylaminoethoxychalcone and 4-dimethylamino-4′- dimethylaminoethoxychalcone were highly active, which were equipotent to reserpine with IC_50_-values of 9.0 and 7.7 lM, respectively. Additionally, three compounds synergistically increased the effect of ciprofloxacin on *S. aureus*, of which 4-phenoxy-4′-dimethylaminoethoxychalcone exhibited a fourfold higher activity at 3.13 μg/mL, being twice as potent as reserpine [70]. Additionally, the ability to inhibition of efflux pump was concentration-dependent. Higher molecule concentration increases many-fold inhibition of efflux pump.

Chalcone derivatives, particularly *trans*-3-(1H-indol-3-yl)-1-(4′-benzyloxyphenyl)-2-propen-1-one, 1-(4″-biphenyl)-3- (3′4′-dihydroxyphenyl)-2-propen-1-one, 1-(4″-hydroxy-3″- methylphenyl)-3-(4′-hydroxyphenyl)-2-propen-1-one, 3-(4′–chlorophenyl)-1-(4″-hydroxyphenyl)-2-propen-1-one and LTG-oxime showed effect on clinical isolates of MRSA by modulating the bacterial efflux pump. These compounds exhibit synergistic interaction with antibiotics– norfloxacin under both in vitro and in vivo conditions [71]. In short, chalcone and its derivatives exhibited antibacterial actions via various targets, which mainly include efflux pump inhibitory (EPI), interfering DNA replication, and filamentous temperature-sensitive mutant Z (FtsZ).

Condensed pyrimidine derivatives, ring-fused chalcones, and flavanones were also reported to have antibacterial activity. In treating certain oral infections caused by bacteria, chalcones were included along with the antibiotics, which were found to enhance the effects of the antibiotics. Rhodanine derivatives have strong antimicrobial activity against methicillin-resistant and non-methicillin-resistant *S. aureus* strains. Quinoxalines derivatives inhibited bacterial activity against the gram-positive bacteria, *Bacillus subtilis*, and *Staphylococcus aureus* [72].

### 3.3. Anti-Fungal Agent

Chalcones act on glutathione, cysteine molecules, and proteins involved in the cell separation of yeast cells. A hybrid molecule of pharmacophore of fluconazole and chalcone was tested for their antifungal activity and showed inhibitory activity against *Candida albicans*. Chalcone with unsubstituted thiophene ring B and thiomethyl substitution at the *p*-position of ring A (compared to *o*- and *m*-) produced credible antifungal activity on fluconazole-sensitive and fluconazole-resistant strains of yeast [73].

The highest antifungal efficacy against standardized strains of *Trichophyton rubrum* was demonstrated by the chalcone 3-(2-chlorophenyl)-1-(2′-hydroxy-4′,6′-dimethoxyphenyl)prop-2-en-1-one (MIC = 12.5 μg/mL), which also inhibited all ten clinical isolates of *T. rubrum* (IC_50_ 12.5 and IC_90_ 25 μg/mL). The *Neurospora crassa* assay revealed a blotchy look in the inhibitory halo formed by this chalcone, strongly indicating that it may work by inhibiting the fungal cell wall [74]. This study also demonstrated that noticeable hyphal curling was observed in the hazy zone at a magnification of 400 or less; therefore, this chalcone appears to be a hyphal deformity inducer.

The highest antifungal activity (MIC: 1.95 μg/mL) was shown by the compounds **62**, **63**, **68**, and **69** against *C. albicans* (Table 2), followed by the compounds **66**, **67** (MIC: 3.90 μg/mL), and compound **65** has shown the same activity (MIC: 15.62 μg/mL) as Amphotericin-B. The remaining compounds have shown lower activity (MIC ≥ 125–62.50 μg/mL) against *C. albicans* [58]. In comparison to *C. albicans*, all compounds were less active against *A. niger*. In this case, compounds **66** and **67** have shown comparatively better antifungal activity (MIC: 7.81 μg/mL) against *A. niger*. However, as compared to Amphotericin-B, compounds **61**, **65** (MIC: 15.62 μg/mL), **68** (MIC: 31.25 g/mL), and **63**, **64** (MIC: 62.50 μg/mL) have shown good antifungal activity against *A. niger* (Table 2). Compounds 66 and 67 have demonstrated equal antifungal activity with Nystatin against *A. niger*, *possibly* due to the presence of a high electron releasing group (Tri-OMe) or without substitutions on the aromatic ring. Similarly, compounds **62**, **63**, and **69** were most active against *C. albicans* as compared to standard drugs Amphotericin-B and Nystatin may be due to the presence of the electron-withdrawing group.

Other observations included significant antifungal activity by fluoro-substitution at the *p*- position on ring A and chalcone compounds with smaller halogen sizes [73]. Several studies on the antifungal activity of chalcones have results that contradict one another. However, they have a common finding: placing the hydroxyl group at the *m*-position in ring A produces a significant antifungal effect [54].

### 3.4. Anti-Malarial Agent

Numerous studies have been conducted to evaluate the antimalarial activity of chalcone and its derivatives for decades to develop novel, safe, less toxic, and highly active antimalarials [75,76,77,78,79,80,81,82,83,84,85,86,87,88]. Among the many chalcones-chloroquinoline hybrid compounds, 3-(4-[1-(7-Chloro-quinolin-4-yl)-1H-[1,2,3]triazol-4-yl-methoxy]-3-methoxy-phenyl)-1-(2,4-dimethoxy-phenyl)-propenone was the most active against different isolates, chloroquine-sensitive (CQS) strain D10 (IC_50_ 0.04 µM) and chloroquine-resistant (CQR) strains Dd2 (IC_50_ 0.07 µM) and W2 (IC_50_ 0.09 µM) of *Plasmodium falciparum* [81].

A series of chalcone derivatives were investigated to determine their antimalarial activity on *P. falciparum* cysteine protease; the results showed varying antimalarial activity depending on the steric and hydrophobic properties, molar refractivity, and molecular length against chloroquine-resistant *P. falciparum*, and the molecular weight against mefloquine-resistant strains [89]. In another study, phenylurenyl chalcone derivatives were synthesized and tested as inhibitors against a chloroquine-resistant strain of *P. falciparum* to evaluate the activity of the cysteine protease falcipain-2, globin hydrolysis, β-hematin formation, and murine *P. berghei* malaria [75]. Among the tested Chalcones, the most active antimalarial compound was 1-[3′-*N*-(*N*′-phenylurenyl) phenyl]-3(3,4,5-trimethoxyphenyl)-2-propen-1-one (IC_50_ of 1.76 μM).

A series of novel keto-enamine Chalcone-chloroquine hybrids were tested against chloroquine-sensitive *P. falciparum* 3D7 strain and found some compounds that exhibited comparable antimalarial activity [76]. Later, highly potent antimalarial compounds were evaluated for their in vivo efficacy using Swiss mice infected by chloroquine-resistant (N-67) strain of *P. yoelii* and demonstrated that compounI(*E*)-2-tert-Butyl-6-((2-(7-chloroquinolin-4-ylamino)ethylamino)methylenI4-((E)-3-(4-fluorophenyl)-3-oxoprop-1-enyl)cyclohexa-2,4-dienone, and (*E*)-4-((E)-3-(4-Bromophenyl)-3-oxoprop-1-enyl)-2-tert-butyl-6-((2-(7-chloroquinolin-4-ylamino)ethylamino)methylene)cyclohexa-2,4-dienone each suppressed 99.9% parasitemia on day 4, possibly by the inhibition of hemozoin formation. This mode of action may be the primary mechanism of action of these compounds against malaria parasites.

A study reported that the C2–C3 double bond is responsible for the high inhibitory activity of chalcones because it links the A and B rings as well as stabilizes the molecular conformation, which allows the drug molecule to bind to the active site more efficiently; there is decreased inhibitory activity by steric interactions due to the substitutions on the bridge portion of chalcone derivatives; increased inhibitory activity was seen in electron-donating substitution on ring A and chloro- or fluoro- substitution on ring B; an increase in antimalarial activity in placing quinolinyl group to ring B [90]. A small hydrophobic nitrogen heterocyclic group at ring B and a small lipophilic functional group at ring A can increase antimalarial activity [90].

### 3.5. Anti-Protozoal and Anti-Filarial Agent

Even though chalcone and its derivatives have diverse biological applications; however, limited applications have been found for antiprotozoal activity [91,92,93]. Since structural modifications of the main pharmacophore of chalcone is the synthesis process of new derivatives, expecting high biological activity of novel compounds is normal. However, the antiparasitic potencies of the α,β-double bond modified chalcones only differ marginally from the potencies of the parent chalcones [93]. This may be limited the further evaluation of chalcone for antiprotozoal activity.

Twenty Chalcone compounds were isolated from plants and tested against extracellular promastigotes of *Leishmania donovani*, L. *infantum*, L. *enrietii* and L. *major*, and against intracellular amastigote L. *donovani* residing within murine macrophages [93]. Most of the compounds were active (EC_50_ 0.07–2.01 µg/mL) against the extracellular *Leishmania* (*L. donovani*). A few chalcones, namely 2′,4′-dihydroxy-4-methoxychalcone, 2′-hydroxy-3,4-dimethoxychalcone and 2-hydroxy-4,4′-dimethoxychalcone (EC_50_ 0.39–0.41 µg/mL) were also significantly inhibited the intracellular survival of L. *donovani* parasites. However, all the compounds showed cytotoxicity (EC_50_ 0.19–2.06 µg/mL) while tested on mammalian macrophages derived from murine bone marrow [92]. Hayat et al. [91] screened fifteen Chalcone molecules, of which only four compounds were found to be more active against the *Entamoeba histolytica*, and one compound was moderately active compared to reference drug metronidazole (IC_50_: 1.46 μM). In addition, all the tested compounds were non-toxic against the human breast cancer MCF-7 cell line (IC_50_: 1.56–50 μM).

TIe chalcIne of interest in having anti-leishmanial activities is licochalcone A; it modifies the structure and function of the *Leishm’nia braziliensis*’s mitochondria as well as the activity of mitochondrial dehydrogenases. In addition, the aforementioned chalcone prevents the promastigotes and amastigotes, which are produced by the parasite, from developing. There is also a decrease in parasite load inside the liver and spleen [94]. Another protozoon called *Trypanosoma cruzi* was used to study the effects of chalcone. The chalcones with no substitution groups exhibit anti-trypanosomal activity [95].

Chalcones were tested on *Setaria cervi* extracted from female adults for anti-filarial effects by inhibiting glutathione-S-transferase (GST) enzyme. There was a significant inhibitory activity of GST, which led to irreversible inhibition of the viability and motility of the parasites, as well as reduced glutathione levels. Results indicated that lipophilic groups of the ring in chalcone containing oxygen or nitrogen were behind the anti-filarial effects, where methoxy groups exhibited the most significant activity [96].

### 3.6. Anti-Inflammatory Agent

The main component of chalcone that contributes to its anti-inflammatory property is the α,β-unsaturated carbon skeletal system. Polymethoxychalcones showed potent inhibitory effects on inflammation, mainly through the production of high IL-1 β-induced PGE_2_, prostaglandin inhibitors, inflammatory cytokines, and normal human epidermal keratinocyte inhibitors. A series of pyran-fused chalcones and trichalcones can inhibit the NF-κB signaling complement system involved in inflammation. At the same time, some compounds showed better inhibitory activity against lipoxygenase than the standard drug indomethacin. Inflammatory cytokines, particularly TNF-α, IL-1β, IL-6, and IL-8 production, were inhibited by chalcones with heterocyclic systems in their moiety. Chalcones converted to Mannich bases also inhibit the release of inflammatory mediators from mast cells, macrophages, neutrophils, and microglial cells. Other chalcone derivatives, such as dihydroxychalcones, also exhibit anti-inflammatory activity [46].

In inflammatory diseases, nardoaristolone A and isobava chalcone significantly inhibited pain-related acute and chronic inflammation and treated rheumatoid arthritis. Other chalcone compounds can also be used to suppress secondary tumor formation and treat rheumatoid arthritis and osteoarthritis. SGCH 19 (1-(7-chloro-3-methyl-1-phenyl-2-naphthyl)-3-(2-furyl)-2-propen-1-one), SGCH 20 (1-(7-chloro-3-methyl-1-phenyl-2-naphthyl)-3-(2-thienyl)-2-propen-1-one), and chalcone compounds with fluoride or chloride groups have a high potency as an anti-inflammatory agent, and may be more potent than the reference drugs, indomethacin and ibuprofen. It was concluded that electron-withdrawing groups (EWG) are responsible for the increased anti-inflammatory activity of chalcones [97].

The anti-inflammatory activity also depends on the position of the substituted group on the phenyl ring A and increases in the order: −3, 4-(OH)_2_, −4-OH, −3,4-OCH_2_O^−^, −3-OCH_3–_4-OH, −4-OCH_3_, −4-N(CH_3_)_2_. Chalcones with fluoride or chloride groups also show potent anti-inflammatory activity, with 4-Cl and 3-NO_2_ being more powerful than 2, 4-Cl_,_ and 2-NO_2_, respectively [2].

Three enzymes were tested with the inhibitory activity of chalcone derivatives on these enzymes, namely the mammalian alpha-amylase, cyclooxygenase (COX), and monoamine oxidases (MAOs) [98,99,100]. In a study involving porcine pancreatic alpha-amylase, trans-chalcones act as a competitive inhibitor by interacting with Trp59 and Tyr62 [101].

### 3.7. Anti-Cancer Agent

In recent years, numerous studies evaluated and revealed the anticancer potential of chalcone and chalcone-based different types of derivatives through in vitro, in vivo as well as molecular docking studies [102,103,104,105,106,107,108,109,110,111]. Both in vitro and in vivo methods were used to explore the anti-melanoma effects of flavokawain B on human melanoma cells and the processes of cell death that were mediated by the generation of reactive oxygen species (ROS) [108]. The findings show that flavokawain B decreased the viability of human melanoma cells as well as the expression of the B-Raf proto-oncogene, serine/threonine kinase (BRAF), and extracellular signal-regulated kinase. The execution of apoptosis was aided by Caspase-3 activation, the PARP cleavage pathway, and Bcl2-associated X (Bax)/B-cell lymphoma 2 (Bcl-2) dysregulation. Additionally, flavokawain B also inhibited tumor growth in nude mice with xenografts [108]. This study concluded that flavokawain B could potentially be used for managing human melanoma cancer by executing and producing ROS-modulated apoptotic and autophagic cell death.

Chouiter et al. [105] synthesized novel chalcone derivatives and tested for their anticancer activity using human lung (A549) and stomach (AGS) cancer cell lines and evaluated them in the noncancer human lung fibroblast (MRC-5) cell lines. All the cell lines tested showed no toxicity for 2-pyrazolines, although the AGS cell line was hazardous for chalcones containing a benzimidazole moiety. Mechanistic studies revealed that these substances cause loss of cell viability and mitochondrial membrane potential while inducing morphological characteristics consistent with regulated cell death by caspase activation, particularly by caspase-3. Boronic chalcones were investigated for anticancer activity, and results indicated the chalcones, particularly AM114, produced a cytotoxic effect in cancer cells by inhibiting the chymotrypsin-like activity of the 20S proteasome in these cells, which in turn accumulated p53 and p21 proteins, creating a favorable condition for cell apoptosis [112]. In other studies, the double bond was replaced with a thiophene ring, which showed the antiproliferative activity of cancer cells and the growth inhibition activity of cancer cell lines at nanomolar to low micromolar concentrations. Some studies have shown inhibition of tubulin polymerization by binding [3H] colchicine to tubulin and arresting the G2/M phase of K562 cells [113,114]. 2′-hydroxychalcone was studied for their antitumor effects on HepG2 hepatocellular carcinoma cells, and results showed they initiate cell apoptosis and inhibit cell proliferation. This was due to the ‘ring A,’ which does not contain methoxy groups. Other chalcones promoted cell apoptosis and cytotoxicity in prostate cancer cells through tumor necrosis factor-related apoptosis-inducing ligand (TRAIL) [114].

In addition, studies conducted on the cytotoxic activity of chalcones to breast cancer cell lines MCF-7 and T47D showed significant cytotoxicity, especially the derivatives containing nitro‘groups, such as ‘*N*-4-hydroxy-3-(3-(2/3/4-nitrophenyl)-acryloyl)’phenyl acetamide’. Another study on quinolinyl chalcone derivatives discovered the potent anti-cancer activity of up to 103% by SGCH 3 (3-(4-chlorophenyl)-1-(3-methyl-1-phenyl-2-naphthyl)-2-propen-1-one) [97]. The coumarinyl-chalcone derivatives (3 h and 3 m) exhibit cytotoxicity towards human cell lines of the lungs, breast, and blood cells, albeit less potent than the standard drug, Imatinib [113,115].

## 4. Conclusions

This review summarized the recent antimicrobial and other pharmacological activities of chalcone compounds with their mode of action. Moreover, we have discussed different methods of chalcone derivatives synthesis and characterization. Notably, chalcone derivatives demonstrated potential antimicrobial activities, including antibiotic-resistant strains like MRSA, KPC, and NDM. Chalcone derivatives potentially inhibit the different targets pathway of microbial resistance. Several chalcone compounds demonstrated optimum characteristics of an effective antimicrobial agent. However, their potential must be warranted using preclinical and clinical studies. Chalcone derivatives can also be a lead compound with anti-inflammatory and anticancer properties, but further study is required to corroborate its clinical activities. Since structure-activity has a potential relationship, even though some modifications revealed negligible improvement, further study is needed for more extensive evaluation, including molecular mechanisms of actions. Even though a plethora of health benefits are evident from experimental studies, evaluating the pharmacological activities of chalcone and its derivatives is still required, particularly in preclinical and clinical studies. As chalcone and its derivatives can be synthesized in the laboratory and chemical structure can be modified, more effective action against a specific disease pathway can be achieved.

## Figures and Tables

**Table 1:** Antibacterial activity of the different types of chalcone and its derivatives [57,59,61,62].

No.	Materials Tested	Antimicrobial Assay	Test Microorganism (MIC, µg/mL)				Antibiotic	Antimicrobial Effect
			***S. aureus* ATCC 29213**	***E. faecalis* ATCC 29212**	***E. coli*** **ATCC 25922**	***S. enterica* ATCC 1307**		
**1**	(E)-N-(2-((4-cinnamoylphenyl)amino)-2-oxoethyl)-N,N-dimethyloctan-1-aminium chloride	BMD	1	2	3	4	VAN, MEM	Strong
**2**	(E)-N-(2-((4-(3-(3-chlorophenyl)acryloyl)phenyl)amino)-2-oxoethyl)-N,N dimethyloctan-1-aminium chloride	BMD	2	2	4	8	VAN, MEM	Strong
**3**	(E)-N-(2-((4-(3-(3-chlorophenyl)acryloyl)phenyl)amino)-2-oxoethyl)-N,N-dimethyloctan-1-aminium chloride	BMD	2	1	4	4	VAN, MEM	Strong
**4**	(E)-N-(2-((4-(3-(4-fluorophenyl)acryloyl)phenyl)amino)-2-oxoethyl)-N,N-dimethyloctan-1-aminium chloride	BMD	2	2	2	4	VAN, MEM	Strong
**5**	(E)-N-(2-((4-(3-(3-fluorophenyl)acryloyl)phenyl)amino)-2-oxoethyl)-N,N-dimethyloctan-1-aminium chloride	BMD	32	64	>128	>128	VAN, MEM	Not good
**6**	(E)-N-(2-((4-(3-(2-fluorophenyl)acryloyl)phenyl)amino)-2-oxoethyl)-N,N-dimethylbutan-1-aminium chloride	BMD	16	16	64	128	VAN, MEM	Good except *S. enterica*
**7**	(E)-N-(2-((4-(3-(2-fluorophenyl)acryloyl)phenyl)amino)-2-oxoethyl)-N,N-dimethyloctan-1-aminium chloride	BMD	0.5	1	2	4	VAN, MEM	Strong
**8**	(E)-N-(2-((4-(3-(2-fluorophenyl)acryloyl)phenyl)amino)-2-oxoethyl)-N,N-dimethyldodecan-1-aminium chloride	BMD	4	8	16	32	VAN, MEM	Good
**9**	(E)-N-(2-((4-(3-(2-fluorophenyl)acryloyl)phenyl)amino)-2-oxoethyl)-N,N-dimethyltetradecan-1-aminium chloride	BMD	8	>128	>128	>128	VAN, MEM	Effective against *S. aureus* only
**10**	(E)-N-(2-((4-(3-(2-fluorophenyl)acryloyl)phenyl)amino)-2-oxoethyl)-N,N-dimethyloctadecan-1-aminium chloride	BMD	>128	>128	>128	>128	VAN, MEM	No activity
**11**	(E)-N-(2-((4-(3-(2,3-difluorophenyl)acryloyl)phenyl)amino)-2-oxoethyl)-N,N-dimethyloctan-1-aminium chloride	BMD	0.5	2	2	8	VAN, MEM	Strong
**12**	(E)-N-(2-((4-(3-(2,4-difluorophenyl)acryloyl)phenyl)amino)-2-oxoethyl)-N,N-dimethyloctan-1-aminium chloride	BMD	2	2	4	4	VAN, MEM	Strong
**13**	(E)-N-(2-((4-(3-(2,6-difluorophenyl)acryloyl)phenyl)amino)-2-oxoethyl)-N,N-dimethyloctan-1-aminium chloride	BMD	32	32	128	128	VAN, MEM	Fair
**14**	(E)-N-(2-((4-(3-(2-ethoxy-5-nitrophenyl)acryloyl)phenyl)amino)-2-oxoethyl)-N,N-dimethyloctan-1-aminium chloride	BMD	8	2	>128	>128	VAN, MEM	Strong against *S. aureus* and *E. faecalis*
**15**	(E)-N-(2-((4-(3-(4-(tert-butyl)phenyl)acryloyl)phenyl)amino)-2-oxoethyl)-N, N-dimethyloctan-1-aminium chloride	BMD	2	4	8	16	VAN, MEM	Strong
**16**	(E)-N,N-dimethyl-N-(2-oxo-2((4(3(2(trifluoromethyl)phenyl)acryloyl)phenyl)amino)ethyl)octan-1-aminium chloride	BMD	2	4	16	16	VAN, MEM	Strong
**17**	(E)-N,N-dimethyl-N-(2-oxo-2-((4-(3-(p-tolyl)acryloyl)phenyl)amino)ethyl)octan-1-aminium chloride	BMD	1	1	4	8	VAN, MEM	Strong
**18**	(E)-N-(2-((4-(3-(4-methoxyphenyl)acryloyl)phenyl)amino)-2-oxoethyl)-N,N-dimethyloctan-1-aminium chloride	BMD	1	2	4	4	VAN, MEM	Strong
**19**	(E)-N,N-dimethyl-N-(2-((4-(3-(naphthalen-2-yl)acryloyl)phenyl)amino)-2-oxoctan-1-aminium chloride	BMD	2	2	8	8	VAN, MEM	Strong
**20**	(E)-N,N-dimethyl-N-(2-oxo-2-((4-(3-(pyridin4yl)acryloyl)phenyl)amino)ethyl)butan-1-aminium chloride	BMD	128	128	>128	>128	VAN, MEM	Not good
**21**	(E)-N,N-dimethyl-N-(2-oxo-2-((4-(3-(pyridin-4yl)acryloyl)phenyl)amino)ethyl)octan-1-aminium chloride	BMD	4	8	16	32	VAN, MEM	Good
**22**	(E)-N,N-dimethyl-N-(2-oxo-2-((4-(3-(pyridin-3-yl)acryloyl)phenyl)amino)ethyl)octan-1-aminium chloride	BMD	4	8	16	32	VAN, MEM	Good
**23**	(E)-N,N-dimethyl-N-(2-oxo-2-((4-(3-(pyridinedin-2yl)acryloyl)phenyl)amino)ethyl)octan-1-aminium chloride	BMD	4	8	16	32	VAN, MEM	Good
**24**	(E)-N-(2-((4-(3-(6-bromopyridin-2-yl)acryloyl)phenyl)amino)-2-oxoethyl)-N, N-dimethyloctan-1-aminium chloride	BMD	4	8	16	32	VAN, MEM	Good
**25**	(E)-((2-((4-(3-(3-fluoropyridin-2-yl)acryloyl)phenyl)amino)-2-oxoethyl)(methyl)(octyl)-l4-azanyl)methylium chloride	BMD	2	8	8	16	VAN, MEM	Strong
**26**	(E)-N-(2-((4-(3-(furan-2-yl)acryloyl)phenyl)amino)-2-oxoethyl)-N,N-dimethyloctan-1-aminium chloride	BMD	1	4	4	16	VAN, MEM	Strong
**27**	(E)-N,N-dimethyl-N-(2-oxo-2-((4-(3-(thiophen-2-yl)acryloyl)phenyl)amino)ethyl)butan-1-aminium chloride	BMD	0.5	1	4	8	VAN, MEM	Strong
**28**	(E)-N, N-dimethyl-N-(2-oxo-2-((4-(3-(thiophen-2-yl)acryloyl)phenyl)amino)ethyl)octan-1-aminium chloride	BMD	32	32	64	128	VAN, MEM	Fair
**29**	(E)-N,N-dimethyl-N-(2-oxo-2-((3-(3-(thiophen2-yl)acryloyl)phenyl)amino)ethyl) octan-1-aminium chloride	BMD	2	4	16	16	VAN, MEM	Strong
			***S. aureus*** ATCC 25923	***B. cereus*** ATCC 11778	***E. coli*** *ATCC 25922*	***P. aeruginosa*** ATCC 27853		
**30**	2,2′,4,4′,5,5′-hexamethoxychalcone	BMD	>2000	>2000	>2000	2000	TET	Not active
**31**	2′-hydroxy-4,4’,5′-trimethoxychalcone	BMD	2000	1000	2000	1000	TET	Not active
**32**	3,4-methylenedloxy-2′-3′,4′,6′-tetramethoxychalcone	BMD	>2000	2000	2000	2000	TET	Not active
**33**	4,4′-dimethoxychalcone	BMD	>2000	>2000	>2000	2000	TET	Not active
**34**	3’,4′-dimethoxychalcone	BMD	1000	1000	2000	1000	TET	Not active
**35**	2-hydroxy’-3’,4′-dimethoxychalcone	BMD	1000	1000	1000	1000	TET	Not active
**36**	2′-acetoxy-3′-4′,4’,6′-tetramethoxychalcone	BMD	2000	2000	1000	1000	TET	Not active
**37**	2,3′,4,4′,5-pentamethoxychalcone	BMD	2000	2000	1000	2000	TET	Not active
**38**	2,2′,4′,5-tetramethoxychalcone	BMD	1000	250	2000	2000	TET	Active against *B. cereus*
**39**	2,3,3′,4,4′,6-hexamethoxychalcone	BMD	2000	2000	2000	2000	TET	Not active
**40**	Cordoin	BMD	2000	2000	2000	2000	TET	Not active
**41**	4-hydroxycordoin	BMD	2000	1000	2000	2000	TET	Not active
**42**	Isocordoin	BMD	2000	2000	2000	2000	TET	Not active
**43**	4-hydroxyisocordoin	BMD	31.2	31.2	1000	1000	TET	Strong for *S. aureus* and *B. cereus*
**44**	Derricin	BMD	2000	2000	2000	2000	TET	Not active
**45**	2-hydroxyderricin	BMD	2000	2000	2000	2000	TET	Not active
**46**	3-hydroxyderricin	BMD	2000	1000	2000	2000	TET	Not active
**47**	4-hydroxyderricin	BMD	7.8	3.9	2000	2000	TET	Strong
**48**	4-methoxyderricin	BMD	2000	2000	2000	2000	TET	Not active
**49**	2′,4,4′-trihydroxychalcone	BMD	62.5	62.5	2000	2000	TET	Strong
**50**	2’, 4,4’-trihydroxy-3-preny’-3’geranylchalcone	BMD	31.2	15.6	1000	1000	TET	Strong
**51**	2’, 4,4’-trihydroxy’-3’geranylchalcone	BMD	31.2	15.6	1000	1000	TET	Strong for *S. aureus* and *B. cereus*
**52**	4-hydroxyisolonchocarpin	BMD	1000	1000	2000	2000	TET	Not active
**53**	Lonchocarpin	BMD	2000	2000	1000	2000	TET	Not active
**54**	4-hydroxylonchocarpin	BMD	2000	2000	2000	2000	TET	Not active
**55**	4-hydroxyisolonchocarpin	BMD	2000	1000	2000	2000	TET	Not active
**56**	Isolonchocarpin	BMD	2000	2000	1000	1000	TET	Not active
**57**	4-hydroxy-4′-methoxychalcone	BMD	500	500	2000	1000	TET	Not active
**58**	2-hydroxydihydrochalcone	BMD	2000	2000	2000	2000	TET	Not active
**59**	2’,4,5′-trihydroxy-3,4-methylene-dioxy-dihydrochalcone	BMD	2000	2000	2000	2000	TET	Not active
**60**	2’,4,5′-trihydroxy-dihydrochalcone	BMD	250	250	>2000	2000	TET	Active against *S. aureus* and *B. cereus*
			***S. aureus***	***B. subtilis***	***S. typhimurium***	***P. aeruginosa***		
**61**	1,3-Bis(4-chlorophenyl)-3-(phenylsulfonyl)propan-1-one	BMD	250	15.62	125	125	AMP, KMN	Strong
**62**	1-Phenyl-3-(4-chlorophenyl)-3-(phenylsulfonyl)propane-1-one	BMD	125	62.5	125	62.5	AMP, KMN	Strong
**63**	1-(4-Chlorophenyl)-3-(3-nitrophenyl)-3-phenylsulfonylprop-ane-1-one	BMD	125	62.5	62.5	62.5	AMP, KMN	Strong
**64**	1-(4-Bromophenyl)-3-phenyl-3-(phenylsulfonyl) propane-1-one	BMD	125	62.5	250	62.5	AMP, KMN	Strong
**65**	1-(4-Bromophenyl)-3-(3,4-dimethoxyphenyl)-3-(phenylsulfonyl)propane-1-one	BMD	62.5	62.5	1.95	62.5	AMP, KMN	Strong
**66**	1-(4-Bromophenyl)-3-(3,4,5-trimethoxyphenyl)-3-(phenylsulfonyl) propane-1-one	BMD	125	31.25	1.95	125	AMP, KMN	Strong
**67**	1-Phenyl-3-phenyl- 3-phenylsulfonylpropane-1-one	BMD	62.5	31.25	1.95	125	AMP, KMN	Strong
**68**	1,5-Di(4-methylphenyl)-1,5-bis(phenylsulfonyl)pentan-3-one	BMD	62.5	62.5	31.25	62.5	AMP, KMN	Strong
**69**	1,5-Di(4-chlorophenyl)-1,5-bis(phenylsulfonyl)pentan-3-one	BMD	250	31.25	15.62	250	AMP, KMN	Strong
**70**	1,5-Di(phenyl)-1,5-bis(phenylsulfonyl)pentan-3-one	BMD	62.5	125	250	62.5	AMP, KMN	Strong
**71**	1,5-Di(4-methoxyphenyl)-1,5-bis(phenylsulfonyl)pentan-3-one	BMD	250	31.25	62.5	125	AMP, KMN	Strong

MIC: Minimum inhibitory concentration; BMD: Broth microdilution; VAN: Vancomycin (MIC: 2 µg/mL for *S. aureus*); MEM: Meropenem (MIC: <0.125 µg/mL for *E. coli*); TET: Tetracycline (MIC: 1 µg/mL for *S. aureus*, 0.25 µg/mL for *B. cereus*, 2 µg/mL *cereus*, 32 µg/mL for *P. aeruginosa*); AMP: Ampicillin (MIC: 500 µg/mL for *S. aureus*, 7.81 µg/mL for *B. subtilis*, 15.62 µg/mL for *S. typhimurium*, 250 µg/mL for *P. aeruginosa*); KMN: Kanamycin (MIC: 500 µg/mL for *S. aureus*, 250 µg/mL for *B. subtilis*, 125 µg/mL for *S. typhimurium*, 125 µg/mL for *P. aeruginosa*).

**Table 2:** Antifungal activity of chalcone and its derivatives [66].

No.	Materials Tested Drug	Test Assay	Antifungal Strains (MIC μg/mL)		Standard Antifungal	Antifungal Effect
			*A. niger*	*C. albicans*		
**61**	1,3-Bis(4-chlorophenyl)-3-(phenylsulfonyl)propan-1-one	BMD	15.62	31.25	AMP-B, NSN	Very strong
**62**	1-Phenyl-3-(4-chlorophenyl)-3-(phenylsulfonyl)propane-1-one	BMD	125	1.95	AMP-B, NSN	Very strong
**63**	1-(4-Chlorophenyl)-3-(3-nitrophenyl)-3-phenylsulfonylprop-ane-1-one	BMD	62.5	1.95	AMP-B, NSN	Very strong
**64**	1-(4-Bromophenyl)-3-phenyl-3-(phenylsulfonyl) propane-1-one	BMD	62.5	125	AMP-B, NSN	Very strong
**65**	1-(4-Bromophenyl)-3-(3,4-dimethoxyphenyl)-3-(phenylsulfonyl)propane-1-one	BMD	15.62	15.62	AMP-B, NSN	Very strong
**66**	1-(4-Bromophenyl)-3-(3,4,5-trimethoxyphenyl)-3-(phenylsulfonyl) propane-1-one	BMD	7.81	3.9	AMP-B, NSN	Very strong
**67**	1-Phenyl-3-phenyl- 3-phenylsulfonylpropane-1-one	BMD	7.81	3.9	AMP-B, NSN	Very strong
**68**	1,5-Di(4-methylphenyl)-1,5-bis(phenylsulfonyl)pentan-3-one	BMD	31.25	1.95	AMP-B, NSN	Very strong
**69**	1,5-Di(4-chlorophenyl)-1,5-bis(phenylsulfonyl)pentan-3-one	BMD	125	1.95	AMP-B, NSN	Very strong
**70**	1,5-Di(phenyl)-1,5-bis(phenylsulfonyl)pentan-3-one	BMD	125	250	AMP-B, NSN	Strong
**71**	1,5-Di(4-methoxyphenyl)-1,5-bis(phenylsulfonyl)pentan-3-one	BMD	125	125	AMP-B, NSN	Strong

BMD: Broth microdilution; AMP: Amphotericin-B (MIC: 500 μg/mL for *A. niger*, 15.62 μg/mL for *C. albicans*), NSN: Nystatin (MIC: 7.81 μg/mL for *A. niger* and *C. albicans*).

## Data Availability

All data have been included in the manuscript.
